# Navigating Conflicting Expectations and Devaluation: A Qualitative Study of Home Health Aide Experiences

**DOI:** 10.1093/geroni/igaf122.3976

**Published:** 2025-12-31

**Authors:** Chenjuan Ma, Sora Aikawa, Hillary Dutton, Soo Yeon Kim, Ching Chen

**Affiliations:** New York University, New York, New York, United States; New York University, New York, New York, United States; New York University, New York, New York, United States; New York University, New York, New York, United States; New York University, New York, New York, United States

## Abstract

Home health aides (HHAs) are crucial in supporting individuals aging in place, offering companionship and assistance with daily activities. Despite their vital role, a lack of understanding regarding their work experiences has impeded efforts to address the HHA workforce shortage. This qualitative study explored HHAs’ experiences, their views on roles and responsibilities, and the unique challenges they face. Data were gathered through five focus groups with a total of 47 HHAs and analyzed using a qualitative thematic approach. The analysis identified two main themes and five sub-themes. Theme 1, “Facing Conflicting Expectations Regarding Job Responsibilities,” addresses the inconsistencies HHAs encounter in role expectations from patients, family members, and the interdisciplinary care team. This theme is supported by three sub-themes: 1) Experiencing a discrepancy in role interpretation, 2) Struggling with ambiguous care plans, and 3) Navigating conflicting demands. Theme 2, “Struggling to be Recognized and Respected as Part of the Care Team,” highlights a key dilemma for HHAs: though often seen as the “eyes and ears” of home care patients, they are not always acknowledged as essential members of the care team. This disconnect can have serious implications for patient care. The sub-themes for this section are: 1) Feeling devalued and unappreciated by clients, colleagues, and employers, and 2) Impacting care quality and patient safety. The findings suggest that clearly defined HHA roles and improved communication between HHAs and other care team members are essential for reducing conflict, fostering collaboration, and ensuring quality care and patient safety.

